# Single Cell RNA Sequencing Identifies HSPG2 and APLNR as Markers of Endothelial Cell Injury in Systemic Sclerosis Skin

**DOI:** 10.3389/fimmu.2018.02191

**Published:** 2018-10-01

**Authors:** Sokratis A. Apostolidis, Giuseppina Stifano, Tracy Tabib, Lisa M. Rice, Christina M. Morse, Bashar Kahaleh, Robert Lafyatis

**Affiliations:** ^1^Division of Rheumatology, Department of Medicine, University of Pittsburgh Medical Center, Pittsburgh, PA, United States; ^2^Boston University School of Medicine, Boston, MA, United States; ^3^Division of Rheumatology and Immunology, Department of Medicine, University of Toledo, Toledo, OH, United States

**Keywords:** ScRNA-seq, HSPG2, APLNR, systemic sclerosis, endothelial dysfunction

## Abstract

**Objective:** The mechanisms that lead to endothelial cell (EC) injury and propagate the vasculopathy in Systemic Sclerosis (SSc) are not well understood. Using single cell RNA sequencing (scRNA-seq), our goal was to identify EC markers and signature pathways associated with vascular injury in SSc skin.

**Methods:** We implemented single cell sorting and subsequent RNA sequencing of cells isolated from SSc and healthy control skin. We used t-distributed stochastic neighbor embedding (t-SNE) to identify the various cell types. We performed pathway analysis using Gene Set Enrichment Analysis (GSEA) and Ingenuity Pathway Analysis (IPA). Finally, we independently verified distinct markers using immunohistochemistry on skin biopsies and qPCR in primary ECs from SSc and healthy skin.

**Results:** By combining the t-SNE analysis with the expression of known EC markers, we positively identified ECs among the sorted cells. Subsequently, we examined the differential expression profile between the ECs from healthy and SSc skin. Using GSEA and IPA analysis, we demonstrated that the SSc endothelial cell expression profile is enriched in processes associated with extracellular matrix generation, negative regulation of angiogenesis and epithelial-to-mesenchymal transition. Two of the top differentially expressed genes, *HSPG2* and *APLNR*, were independently verified using immunohistochemistry staining and real-time qPCR analysis.

**Conclusion:** ScRNA-seq, differential gene expression and pathway analysis revealed that ECs from SSc patients show a discrete pattern of gene expression associated with vascular injury and activation, extracellular matrix generation and negative regulation of angiogenesis. HSPG2 and APLNR were identified as two of the top markers of EC injury in SSc.

## Introduction

Vascular injury is a hallmark event in the pathogenesis of Systemic Sclerosis (SSc) (1). Endothelial dysfunction happens early in the course of the disease and drives some of the most prominent clinical manifestations of SSc, including Raynaud's phenomenon (RP), telangiectasias, gastric antral vascular ectasias (GAVE), pulmonary arterial hypertension (PAH), and SSc renal crisis (SRC) (1, 2). Loss of nailfold capillaries, nailfold microhemorrhages, and “giant” capillaries are a very useful tool for physicians to diagnose SSc and underlines the importance of microvascular damage in the disease progression (3). Histopathologic examination of the affected vessels reveals extensive intimal hyperplasia, adventitial fibrosis and vascular smooth muscle hypertrophy that lead to luminal narrowing and ultimately occlusion and thrombosis (4, 5). The result is progressive tissue hypoxia, recurrent cycles of ischemia—reperfusion injury and inflammatory changes. In the majority of SSc patients, vascular changes precede the onset of fibrosis suggesting that endothelial injury is central in the pathogenesis of the disease (6–8) and can involve organs in which fibrosis is not traditionally seen such as the kidneys.

The exact mechanisms that lead to endothelial cell injury and propagate the vasculopathy in SSc are not well understood. The presence of tissue hypoxia should promote compensatory angiogenesis. However, this process is defective in SSc patients who exhibit impaired neovascularization and loss of capillaries and arterioles leading to painful digital ulcerations, PAH and SRC (1, 9, 10). A complex network of interactions between endothelial cells, pericytes, myofibroblasts, and the extracellular matrix (ECM) has been implicated in the pathogenesis of SSc (8). It is currently unclear what drives the activation of fibroblasts and the increased ECM deposition responsible for the fibrotic changes seen in SSc. The endothelial cell injury has been proposed to play a prominent role through the production of activating cytokines by SSc endothelial cells (2, 11), disruption of vascular permeability and extravasation of growth factors (1), induction of hypoxia (2), and possibly by contributing to the pool of myofibroblasts through endothelial-to-mesenchymal transition (12).

Altered gene expression, alternative splicing and epigenetic mechanisms have been shown to contribute to the aberrant endothelial function (13–15). Prior gene expression profiling studies (16–19) and proteome-side analyses (13) have shed light onto the molecular pathways affected in SSc patients. However, these studies do not address the discrete contributions of the implicated cell subsets or individual cells and they do not account for cellular heterogeneity and differential cell composition of the target tissues. Thus, interpretation of their results is limited.

In this report, we implemented single cell sorting and subsequent RNA sequencing of cells isolated from SSc and healthy control (HC) skin. We present evidence that scRNA-seq provides a robust platform for cellular identification that allows for gene expression analysis at the single cell level and accounts for cellular heterogeneity. We focus on skin endothelial cells and define the differential *in situ* gene expression profile in SSc patients. Using pathway analysis software, we highlight the implicated molecular pathways. Finally, we verify independently on skin biopsies using immunohistochemistry and on primary endothelial cells using qPCR that APLNR and HSPG2 represent markers highly expressed in endothelial cells from SSc skin and can potentially be used as surrogates of endothelial dysfunction in SSc patients.

## Materials and methods

### Study participants

The Boston University Medical Center Institutional Review Board (Boston, MA, USA) reviewed and approved the conduct of this study. Informed consent was obtained from patients with diffuse cutaneous SSc [according to diagnostic (20) and subtype (21) criteria] and healthy subjects. Skin biopsies were obtained from the dorsal mid forearm and immediately collected in PBS for single cell isolation. The modified Rodnan skin score (MRSS) was determined for each patient on the day of the biopsy (22).

For the qPCR studies with primary endothelial cells, human microvascular endothelial cells (MVECs) were isolated as described previously (23) from skin biopsies of four diffuse cutaneous SSc patients and four age and sex-matched healthy controls. Informed consent was obtained in compliance with the Institutional Review Board of Human Studies of University of Toledo. All patients fulfilled the American College of Rheumatology criteria for the diagnosis of SSc; they were not on immunosuppressive or steroid therapy and none had digital ulcers or PAH.

### Skin digestion and single cell suspension preparation

Skin digestion was performed using the whole skin dissociation kit for human (130-101-540, Macs Miltenyi Biotec). Enzymatic digestion was completed in 2 h, followed by mechanical dissociation using gentleMacs Dissociator running the gentleMACS program h_skin_01.

### MoFlo analysis

Live cells were stained using NucBlue Live Cell Stain ReadyProbes reagent (Hoechst33342), and sorted using fluorescence-activated cell sorting (FACS) with a Beckman Coulter MoFlo Legacy, excited with multi line UV and detected with 450/20 band pass filter. Cells were deposited with cyclone in TCL buffer (Qiagen) on a 96-well plate, and stored at −80°C until RNA-seq processing.

### RNA-seq protocol and data analysis

RNA-seq was performed using the SmartSeq2 protocol. The SmartSeq2 libraries were prepared according to the SmartSeq2 protocol (24) with some modifications (25). The Smart-Seq2 data was processed at the Broad Institute using a standard computational pipeline. Libraries were barcoded by cell. They were sequenced using Illumina NextSeq platform. Data was deconvoluted by barcode and aligned using Tophat version 2.0.10 (26). Transcripts were quantified using the Cufflinks suite version 2.2.1 (27). Cuffnorm files were analyzed using the R environment for statistical computing (version 3.2.1). Using R, we performed t-distributed stochastic neighbor embedding (t-SNE) analysis, k-means clustering and hierarchical clustering. The following packages were used in R: tsne, rtsne, heatmap.2, rorc, gplots, ggplot2, hmisc, reshape, stringr, mixtools, reshape2, vioplot, seurat. The following parameters were used for t-SNE plots: perplexity 30, max iterations at default of 1000, initial dimensions at 10 and theta 0.0. Pathway analysis was performed using the Gene Set Enrichment Analysis software (GSEA) developed by the Broad Institute (28). Our dataset was compared against the following reference genesets: extracellular matrix, KEGG ECM receptor interactions, hallmark epithelial mesenchymal transition, positive regulation of angiogenesis, negative regulation of angiogenesis. Data was also analyzed with the Ingenuity Pathway Analysis (IPA, QIAGEN Inc., https://www.qiagenbioinformatics.com/products/ingenuity-pathway-analysis).

### MVEC cultures

Microvascular endothelial cells (MVECs) were isolated from the biopsy samples and purified using CD31 magnetic beads as previously described (23) and cultured in Clonetics Endothelial Cell Basal Medium-2 (EBM-2) supplemented with EGM-2-MV growth factors (EGM-2) at 37°C in 5% CO2. Normal control dermal MVECs were similarly derived from healthy adult donors who were matched with the SSc patients for age, sex and race.

### Immunohistochemistry, immunofluorescence, and qPCR

Immunohistochemistry for HSPG2 was performed using an anti-HSPG2 antibody (anti-Perlecan, mouse IgG1 Antibody, clone 5D7-2E4, Millipore Sigma), using a staining protocol as previously described (29). Four healthy control skin biopsies and six scleroderma skin biopsies were stained with anti-HSPG2 antibody. Quantitative real-time PCR was performed using primary endothelial cells (MVECs) for *APLNR* and *ACTB* control, using the ddCT method as previously described (30), with the following TaqMan probes on a 7300 Real-Time PCR system (Applied Biosystems):

*APLNR*: Hs00270873_s1, FAM-MGB, Cat. #4453320 (ThermoFisher Scientific, Applied Biosystems).
*ACTB*: Hs01060665_g1, FAM-MGB, Cat. #4448892 (ThermoFisher Scientific, Applied Biosystems).

Immunofluorescent single and dual antibody staining using tyramide signal amplification (Tyramide SuperBoost Kits with Alexa Fluor Tyramides; ThermoFisher Scientific, Waltham, MA) were performed on formalin fixed paraffin embedded SSc skin tissues. Tissue sections (5 μm thick) were depariffinized and rehydrated followed by heat induced antigen retrieval in citrate buffer pH6.0 (Vector Labs, Burlingame, CA) for 10 min then allowed to cool for 10 min. Blocking was achieved by using 3% H2O2 followed by 10% goat serum (ThermoFisher Scientific, Waltham, MA) for 1 h each. Dual antibody staining was performed using combinations of mouse monoclonal anti-HSPG Perlecan antibody (1:100; Millipore, Temecula, CA); anti-human Von Willebrand Factor mouse monoclonal antibody (1:100; Dako, Santa Clara, CA); anti-human Von Willebrand Factor rabbit polyclonal antibody (1:50); Sigma Aldrich, St. Louis,MO) and Apelin receptor rabbit monoclonal antibody(clone: 5H5L9, 1:500, ThermoFisher Scientific, Waltham, MA). All primary antibodies were incubated overnight at 4°C. Enzymatic development was performed using appropriate goat anti-rabbit or goat anti-mouse Poly-HRP conjugated secondary antibodies (ThermoFisher Scientific, Waltham, MA) for 1 h followed by Alexa Fluor 488 or 594 labeled tyramide solution and completed with reaction stop solution (ThermoFisher Scientific, Waltham, MA) Dual antibody stained samples underwent the same staining process; after the first antibody development was complete tissue again underwent an additional heat induced antigen retrieval, blocking, incubation with compatible primary antibody, poly-HRP secondary antibody and development with spectrally compatible tryamide Alexa Fluor. Slides underwent nuclear staining with Hoeschst stain (1:2000; ThermoFisher Scientific, Waltham, MA). All wash steps consisted of PBS washes 3 times 10 min each. Finally, slides were cover slipped, using ProLong Diamnond Antifade Mountant (ThermoFisher Scientific, Waltham, MA). Images were taken using an Olympus FLUOVIEW FV1000 (Olympus, Waltham, MA) confocal laser-scanning microscope.

## Results

### Dimensionality reduction and clustering of the dataset

In order to discover the altered regulation of gene expression in diffuse cutaneous systemic sclerosis (dcSSc), we analyzed skin biopsies from one dcSSc patient and one age and sex-matched healthy control. Supplementary Figure 1 shows H&E staining of the skin of the dSSc patient with notable fibrosis and inflammatory infiltration. The skin biopsies were digested and single cell suspensions were used to FACS sort single cells in individual wells, which were subsequently used for cDNA library creation and down-stream RNA sequencing. We successfully sequenced 88 cells from the healthy skin biopsy and 96 cells from the SSc skin biopsy.

**Figure 1:**
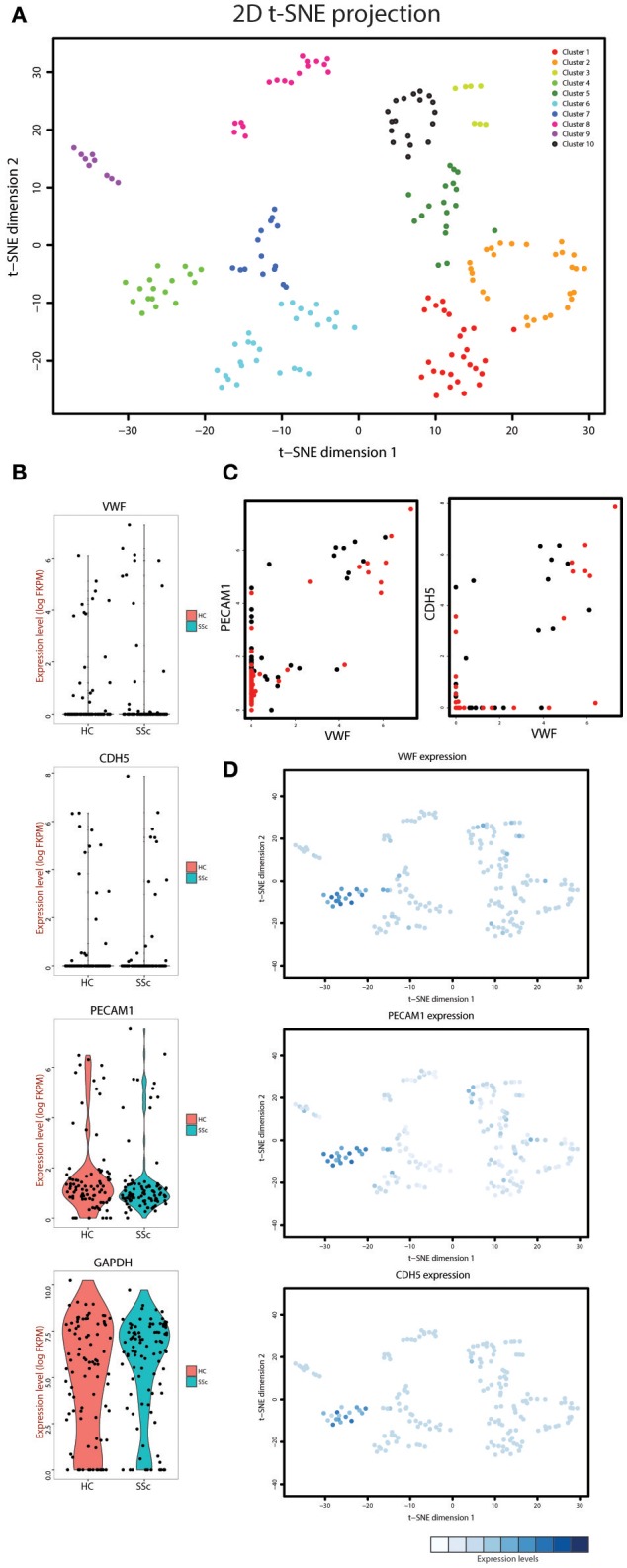
**(A)** t-SNE analysis of the cells isolated from the systemic sclerosis (SSc) and healthy control (HC) skin. Overlaid is k-means clustering with a defined number of 10 clusters with every cluster represented by a different color. **(B)** Violin plots showing the expression levels and density of expression for the *VWF, CDH5, PECAM1*, and *GAPDH* genes in the cells derived from HC and SSc skin. **(C)** Co-expression plots of *VWF* with *PECAM1* (left) and *CDH5* (right) in both HC (black dots) and SSc (red dots) skin cells. **(D)** Overlay of the t-SNE analysis with the expression level of *VWF, PECAM1*, and *CDH5* for each cell. The expression levels for each gene were normalized within the respective graph.

For our initial goal to visualize and ultimately define the various cell subsets in the dataset, we used t-distributed stochastic neighbor embedding (t-SNE), a method of unsupervised learning for dimensionality reduction. 2D projection of the t-SNE effectively reduced the dimensionality of the data, revealing clustering patterns that represent distinct cellular populations (Supplementary Figure 2). Next, we performed K-means clustering analysis of the t-SNE output. First, we calculated the recommended number of k-means centers to be 10 using the “elbow criterion” (Supplementary Figure 3). Subsequently, we overlaid the k-means clustering on the t-SNE projection to visualize individual clusters (Figure 1A).

### Identification of individual cell subpopulations

In order to define the cluster that represents the endothelial cell population in our dataset we employed known endothelial cell markers. Von Willebrand factor (gene name *VWF*), platelet endothelial cell adhesion molecule (gene name *PECAM1*) and vascular endothelial cadherin (gene name *CDH5*) were expressed in a distinct group of cells in our dataset (Figure 1B). Furthermore, plotting *VWF* against *PECAM1* and *CDH5* demonstrated that the cells expressing high levels of *VWF* were also the cells that expressed increased levels of *PECAM1* and *CDH5* (Figure 1C). Next, we overlaid the expression of these genes on the t-SNE projection plot (Figure 1D) and were able to identify cluster 4 as the endothelial cell cluster (see Figure 1 for cluster numbering). This cluster contained 9 cells from healthy control skin and 8 cells from SSc skin. We used the gene expression profiles of these cells for the rest of the analysis.

### Differential gene expression profile of endothelial cells in SSc vs. healthy skin

**Figure 2:**
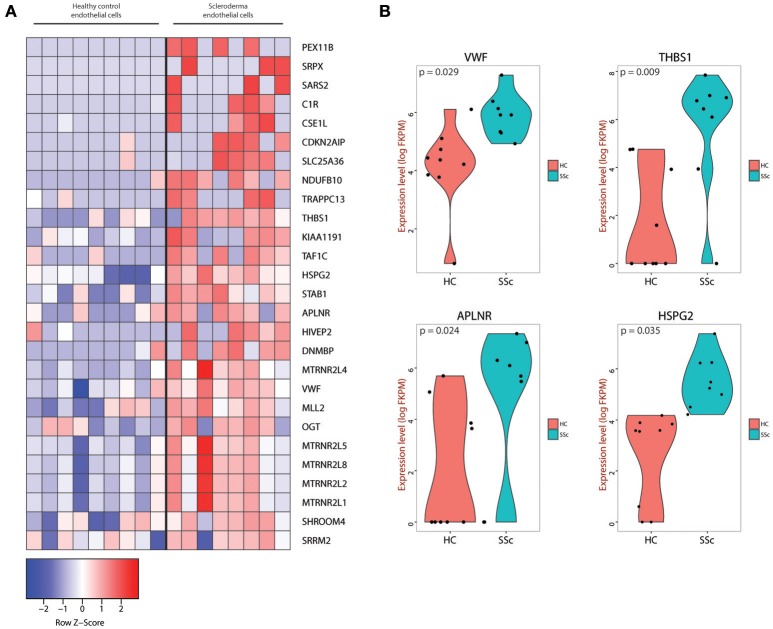
**(A)** Heatmap of genes that were at least two-fold upregulated in SSc endothelial cells compared to endothelial cells isolated from healthy skin. **(B)** Violin plots for the *VWF, THBS1, APLNR, and HSPG2* genes showing the expression level and density of expression for the endothelial cells from HC and SSc skin. The corresponding *p*-values are also depicted for each gene.

After defining the endothelial cells in our dataset, we identified differentially expressed genes between HC and SSc ECs of the skin. We focused on the two-fold up-regulated or two-fold down-regulated genes in SSc compared to HC endothelial cells (Supplementary Figure 4 and Supplementary Table 1). Differential expression was performed using excel. Pair-wise comparison was done with t-test and FDR applied to account for multiple tests. Only genes that that had >2.0 or < −2.0 FC in a statistically significant manner were included. Figure 2A shows the genes upregulated by at least two-fold in a statistically significant manner (top bin) that contain already established markers of endothelial injury and activation, such as the Apelin receptor *APLNR* (31–36), as well as previously identified markers of vascular dysfunction in SSc, such as *THBS1* (16, 37–39) and *VWF* (13, 40,–44). The top bin also includes components of the extracellular matrix, including the heparan sulfate proteoglycan 2 (gene name *HSPG2*) that was previously shown to be implicated in fibrotic processes (45, 46, 47) including SSc-associated fibrosis (48), wound healing (49, 50) and TGF-β signaling (51, 52). Violin plots for the expression of *VWF, THBS1, APLNR*, and *HSPG2* demonstrate that these genes are upregulated in endothelial cells from SSc skin compared to HC skin (Figure 2B).

### Pathway enrichment analysis

**Figure 3:**
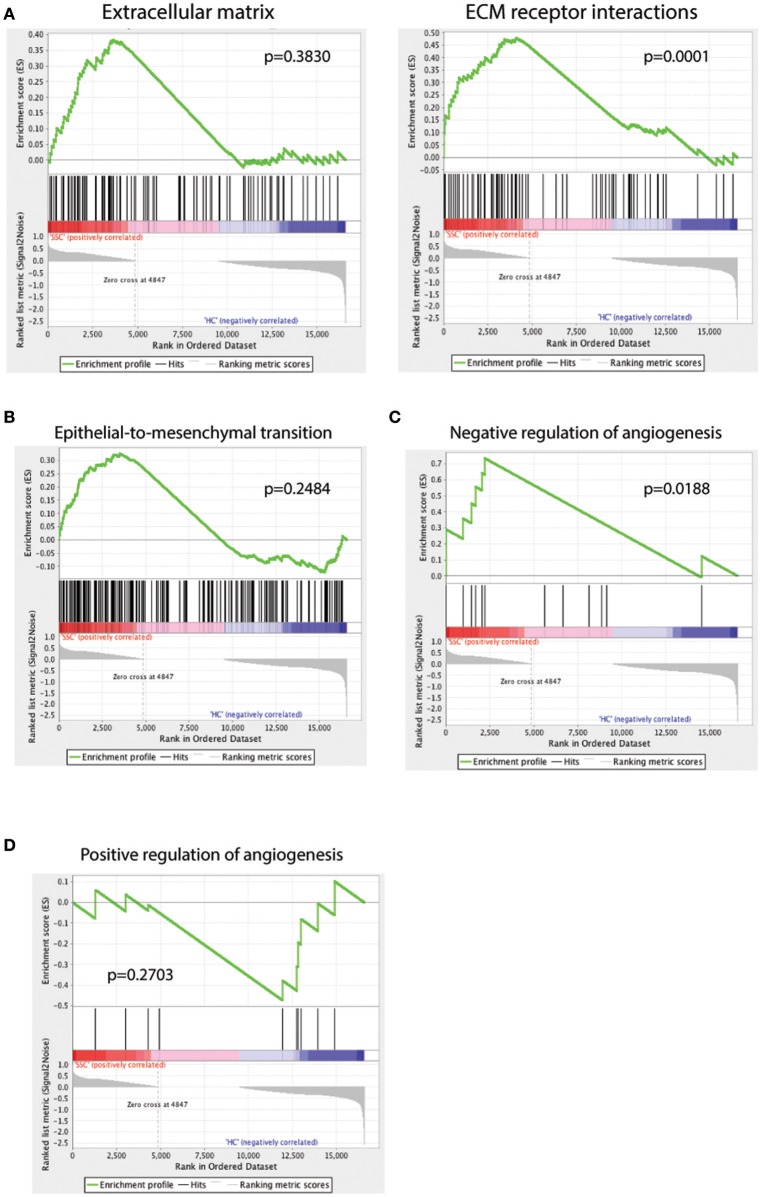
GSEA analysis of the scRNAseq dataset against the GSEA geneset for extracellular matrix (**A**, left) and extracellular matrix (ECM) receptor interactions (**A**, right), epithelial-to-mesenchymal transition **(B)**, negative regulation of angiogenesis **(C)** and positive regulation of angiogenesis **(D)**. A positive enrichment score on the y-axis indicates positive correlation with the SSc endothelial cell group and a negative enrichment score indicates a negative correlation.

In order to understand the pathways enriched in our dataset and to find gene signatures that are positively or negatively regulated in SSc endothelial cells compared to their healthy counterparts, we used Gene Set Enrichment Analysis (GSEA) (28) and Ingenuity Pathway Analysis (IPA, Qiagen). GSEA provides a software platform to analyze gene expression data through pairwise comparison of the dataset of interest with known biological processes and pathways. ECM alterations are a prominent feature in SSc pathogenesis. Intimal thickening and adventitial fibrosis are commonly seen in biopsies of SSc skin and are the pathologic representations of defective angiogenesis, skin fibrosis and vascular wall remodeling. Previous functional studies have shown that endothelial cells play a central role by promoting the fibrotic intimal lesions through interaction with vascular smooth muscle cells and pericytes (1, 53) and possibly by TGF-β mediated endothelial-to-mesenchymal transition (1, 54, 55). Using genesets that represent the ECM production and ECM receptor interactions, we found that the SSc endothelial cell gene expression profile correlated weakly with ECM (*p* = 0.38, Figure 3A, ECM) but more strongly in the complex interactions associated with ECM formation and receptor interactions (*p* = 0.0001, Figure 3A, ECM receptor interaction). Using a signature geneset for epithelial-to-mesenchymal transition (EMT), we show that SSc endothelial cells showed a trend toward enrichment in EMT-associated genes (*p* = 0.284, Figure 3B). Finally, in order to better understand the effect of SSc in angiogenesis, we employed two different GSEA sets: one corresponding to negative and one to positive regulation of angiogenesis. SSc endothelial cells demonstrated enrichment of genes associated with negative regulation of angiogenesis (*p* = 0.018, Figure 3C); conversely, genes associated with the positive regulation of angiogenesis showed a trend toward downregulation in SSc. (*p* = 0.2703, Figure 3D). Thus, GSEA demonstrates that the SSc endothelial cell expression profile is enriched in processes associated with ECM generation, weakly associated with EMT, and detects angiogenesis to be negatively regulated in SSc endothelial cells.

**Figure 4:**
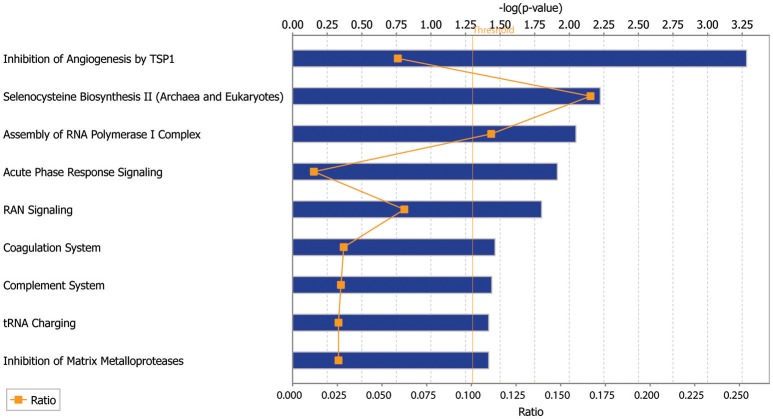
Ingenuity Pathway Analysis (IPA) of the scRNAseq database showing the top canonical pathways enriched in the endothelial cells derived from SSc skin compared to those from healthy control skin.

In order to find the top biologic processes, signatures, and pathways associated with our dataset, we used Ingenuity Pathway Analysis (Qiagen). Examining the canonical pathways enriched in our database, we found the SSc endothelial cells show enrichment in pathways associated with inhibition of angiogenesis, acute phase response, complement activation and matrix metalloproteinases (Figure 4).

### Independent verification of sentinel markers

**Figure 5:**
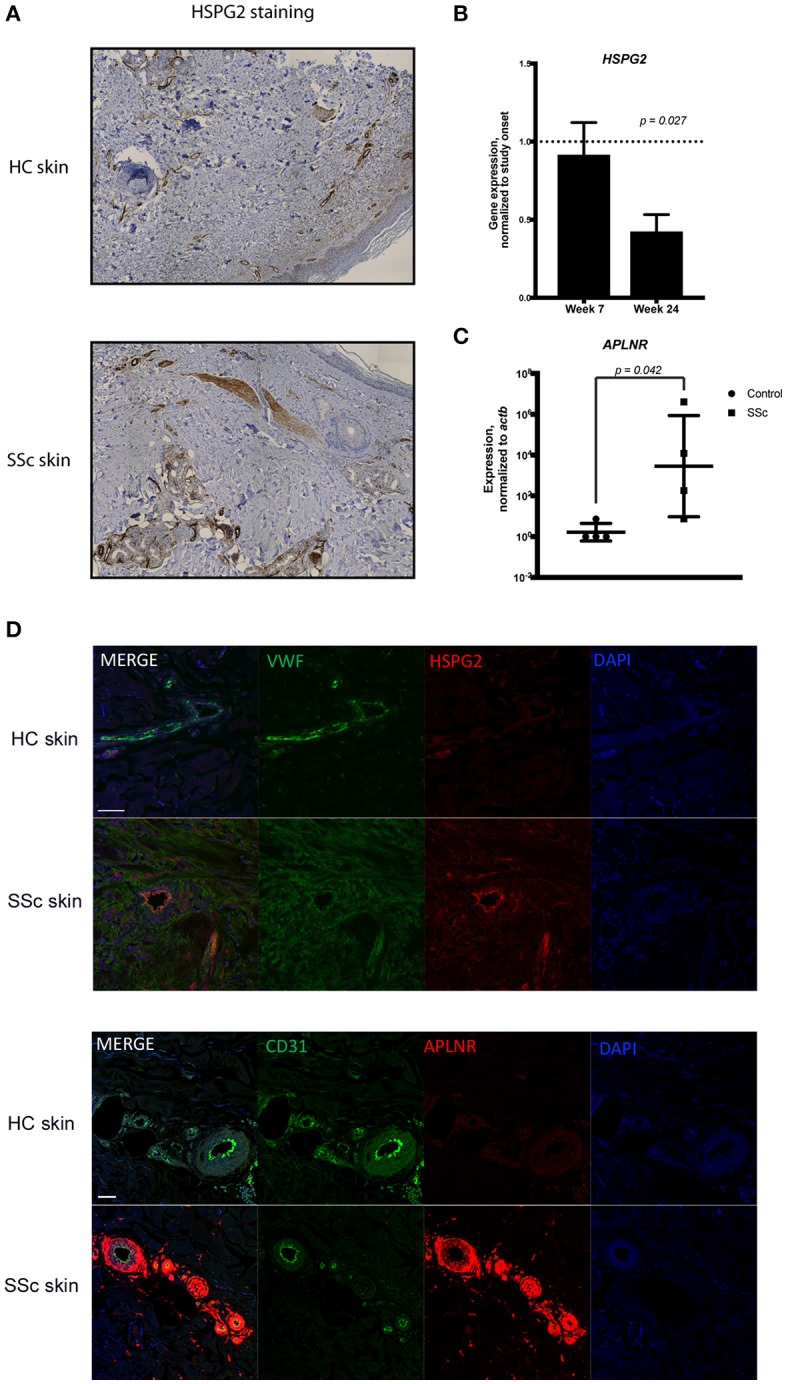
**(A)** Immunohistochemistry of skin biopsies from healthy and SSc skin showing the expression of HSPG2. **(B)** The microarray data from the Fresolimumab clinical trial were analyzed to show the expression of *HSPG2* gene within the study subjects during treatment with fresolimumab at week 7 and week 24. Expression levels are normalized to the onset of study at the time of enrollment. Data were analyzed using ANOVA and shown is the *p-*value. **(C)** qPCR analysis of the expression of *APLNR* in MVECs isolated from healthy and scleroderma skin. Data are normalized to *ACTB. t*-test analysis was performed and the *p*-value is shown. **(D)** Dual antibody immunofluorescent (IF) staining of formalin fixed paraffin embedded scleroderma and control skin biopsies sectioned in 5 μm sections. Upper panels show dual IF staining of HSPG2 (green) and Von Willebrand factor (red) co-localizing on blood vessels. Lower panels show dual IF staining of Von Willebrand factor (green) and APLNR (red) co-localizing on blood vessels in scleroderma skin with DAPI nuclear stain. Scale bar = 50mm.

Complementing our scRNA-seq experiments, we independently verified our findings using distinct experimental protocols. First, we stained skin biopsies of SSc skin and HC skin with the extracellular matrix protein HSPG2, one of the differentially expressed genes in the scRNA-seq experiments (Figure 2). HSPG2 staining was more robust in SSc skin, especially in the perivascular areas (Figure 5A). HSPG2 has been shown to be regulated in a TGF-β dependent manner (51, 52). Both APLNR and HSPG2 co-stained by immunoflorescence with VWF, confirming that they are expressed by endothelial cells in SSc skin (Figure 5D). In order to explore the effect of TGF-β signaling blockade on HSPG2 in SSc patients, we used our previously generated microarray data from our clinical trial of a TGF-β blocking antibody, fresolimumab (for patient characteristics, study details, outcomes and microarray data, refer to (16), Clinicaltrials.gov NCT01284322, GEO database accession number GSE55036). In this trial, SSc patients were treated for 7 and 24 weeks with fresolimumab (Figure 5B) and HSPG2 expression was reduced by the end of 24 weeks in a statistically significant manner (ANOVA, *p* value 0.027), indicating that HSPG2 expression is inhibited by TGF-β blockade, coincident with the decrease in disease activity and skin inflammation seen with fresolimumab.

APLNR was another gene found to be increased in the SSc endothelial cells using scRNA-seq (Figure 2). To independently verify this result, we studied *APLNR* gene expression in microvascular endothelial cells (MVECs) isolated from the skin of four distinct SSc patients and four healthy controls using qPCR. *APLNR* was more highly expressed by SSc primary endothelial cells compared to HC endothelial cells (Figure 5C).

## Discussion

Vascular injury is a central event in the pathophysiology of SSc. However, our knowledge regarding the pathogenesis and pathways involved in the generation of endothelial cell injury is limited. Here, we provide a comprehensive analysis of scRNA-seq data generated from cells derived from SSc and healthy skin. We show that scRNA-seq provides a robust platform for identifying specific cell subtypes and more importantly differential gene expression profiles and signatures in the cell subpopulations of interest at the single cell level.

Using scRNA-seq, we identified distinct markers that can provide insight in the development of endothelial cell injury, including markers that have been previously linked with the pathogenesis of SSc, such as Thrombospondin 1 and Von Willebrand Factor (13, 16, 37–44). Additional genes identified by our analysis, notably *APLNR* and *HSPG2*, have not been previously linked to SSc pathogenesis. These genes are of particular interest as they have been associated with vascular activation and dysfunction as well as fibrosis in different settings (31–36, 45–50, 56).

Apelin and Elabela are both ligands of the Apelin receptor (*APLNR/APJ*), a G-protein coupled receptor found on endothelial cells (57). These ligands play central roles in cardiovascular development and angiogenesis (31, 35, 56, 58). The Apelin/Elabela-APLNR signaling is critical in regulating vascular maturation and APLNR –/– mice are embryonically lethal secondary to cardiovascular defects (33). APLNR expression is induced in conditions of hypoxia (32, 36). APLNR signaling has not been investigated in SSc other than data suggesting that elevated Apelin levels are associated with more severe vascular disease (59). The Apelin/Elabela-APLNR signaling has been implicated in pulmonary arterial hypertension, one of the major complications of SSc (60, 61). In this setting, increased Apelin activity appears beneficial and receptor agonists are being considered as therapies. In this context, increased APLNR expression might represent a positive adaptive response to hypoxia, fibrosis and/or other vascular injury in the SSc skin microenvironment. On the other hand, APLNR has been associated with pathological retinal angiogenesis and telangectasias (34, 62) and, thus, it might contribute to the dysregulated angiogenesis seen in SSc.

The *HSPG2* gene codes for the extracellular matrix protein perlecan, which represents a main component of the blood vessel basement membrane (63). HSPG2 has been implicated in several fibrotic processes, including liver fibrosis and desmoplastic tumors (45–47). Interestingly, a C-terminal fragment of HSPG2 was found to be a main fibrogenic mediator produced by apoptotic SSc endothelial cells (48). Specifically, the C-terminal HSPG2 fragment produced by apoptotic SSc endothelial cells induced PI3K-dependent resistance to apoptosis in fibroblasts and activated myofibroblast differentiation (48). HSPG2-deficient mice exhibit delayed wound healing (49), impaired angiogenesis (49, 52) and decreased TGF-β production in the mouse skin (50). Reciprocally, TGF-β signaling induces HSPG2 promoter activity (51). Analysis of the fresolimumab clinical trial microarray data suggests that *HSPG2* gene expression in SSc skin is regulated by TGF-β. Thus, TGFβ up-regulation of HSPG2 may mediate a profibrotic response to vascular injury in SSc skin.

Our pathway analysis on the single-cell platform demonstrates that the pathways of extracellular matrix generation, inhibition of angiogenesis, epithelial to mesenchymal transition and matrix metalloproteinase production are highly activated and centrally involved in the pathophysiology of endothelial cell injury in SSc patients. A recent study has highlighted the potential importance of endothelial-mesenchymal transition in SSc (64). Our pathway analysis, showing a trend toward upregulated epithelial-mesenchymal transition, supports this notion.

A limitation of the current study is that only one patient and one control biopsy were analyzed by scRNA-seq. However, ancillary studies examining RNA expression in cells and skin immunohistochemistry support the generalizability of the scRNA-seq findings. We anticipate with emerging technologies that more cells and more robust signatures might be found.

Vascular injury occurs early in the disease course of SSc. Raynaud's phenomenon and other vascular malformations can precede the development of overt disease for years to decades. Understanding the endothelial cell injury pathways has the potential of allowing early identification of patients and more accurate prognostication. In addition, our data point to several upregulated endothelial cell genes in SSc skin and implicate these genes in distinct pathways in SSc pathogenesis. Our results provide the framework for the development of biomarkers representing vascular injury and therapeutic targets to be further explored.

## Ethics statement

This study was carried out in accordance with the recommendations of IRB committee of Boston University Medical Center. All subjects gave written informed consent in accordance with the Declaration of Helsinki.

## Author contributions

SA, GS, TT, LR, and CM designed and performed experiments, analyzed the data, and prepared the manuscript. BK analyzed the data and prepared the manuscript. RL designed experiments, analyzed the data and prepared the manuscript.

### Conflict of interest statement

The authors declare that the research was conducted in the absence of any commercial or financial relationships that could be construed as a potential conflict of interest.

## Supplementary material

Supplementary Figure 1 H&E staining of the skin from the dcSSc patients used for the scRNA-seq showing the extensive fibrosis (arrows) and inflammatory infiltration (arrowheads).

Supplementary Figure 2 Two-dimensional projection of the t-SNE analysis of the cells isolated from the systemic sclerosis (SSc) and healthy control (HC) skin. Cells were pooled together to facilitate cell subset identification.

Supplementary Figure 3 The “elbow” criterion used to determine the optimal number of clusters to be used for the k-means clustering used in conjunction with the t-SNE analysis.

Supplementary Figure 4 Heatmap with hierarchical clustering of genes that were at least two-fold up-regulated or down-regulated in SSc endothelial cells compared to endothelial cells isolated from healthy skin.

Supplementary Table 1 Genes regulated < 2-fold in SSc compared to healthy skin endothelial cells.
