# Supplementary figures and images for: Single Cell RNA Sequencing Identifies HSPG2 and APLNR as Markers of Endothelial Cell Injury in Systemic Sclerosis Skin
Source: Front Immunol. 2018 Oct 1;9:2191. doi: 10.3389/fimmu.2018.02191 (PMC6174292; doi:10.3389/fimmu.2018.02191)

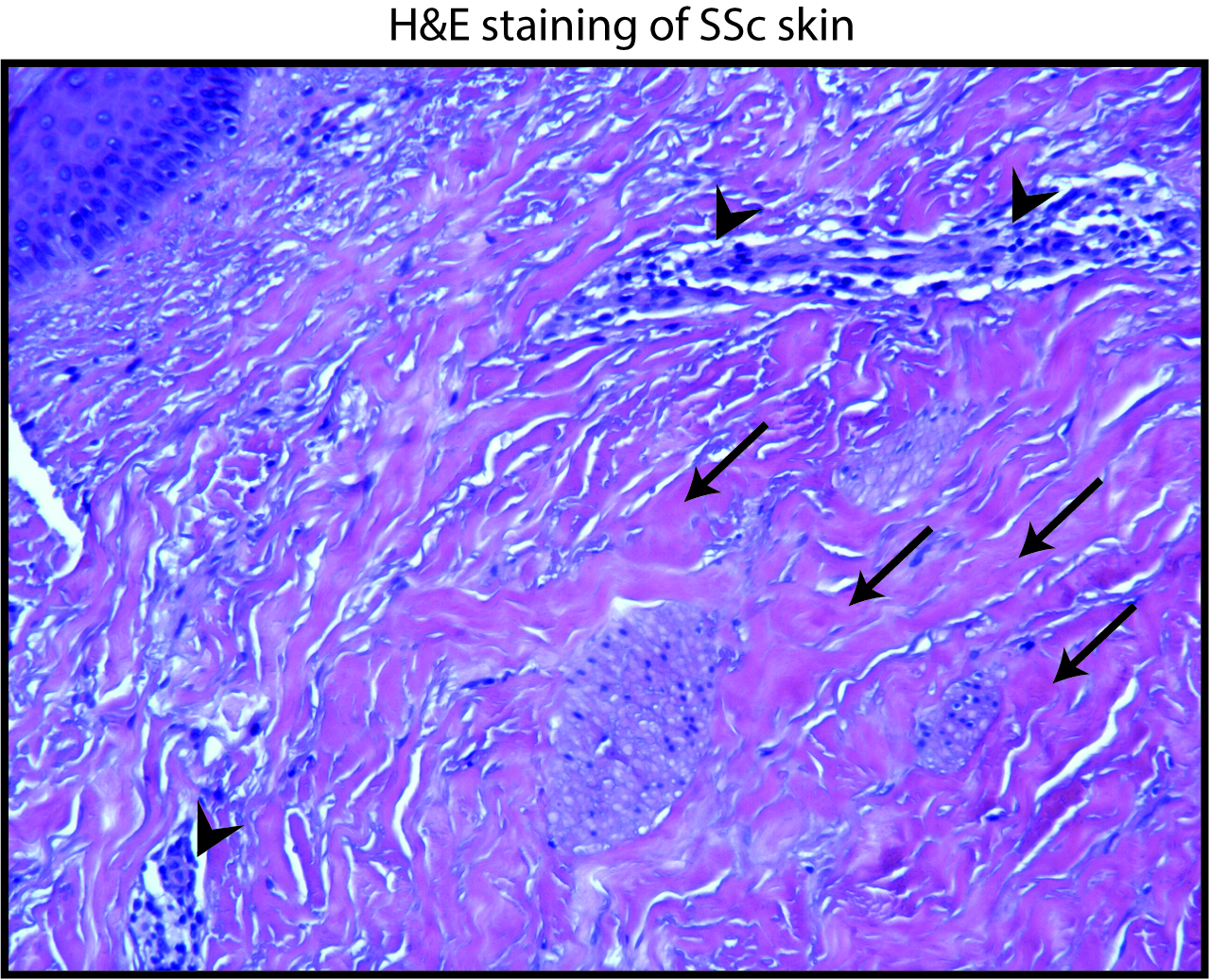

Supplement: Supplementary Figure 1 — H&E staining of the skin from the dcSSc patients used for the scRNA-seq showing the extensive fibrosis (arrows) and inflammatory infiltration (arrowheads). [file Image_1.TIF]

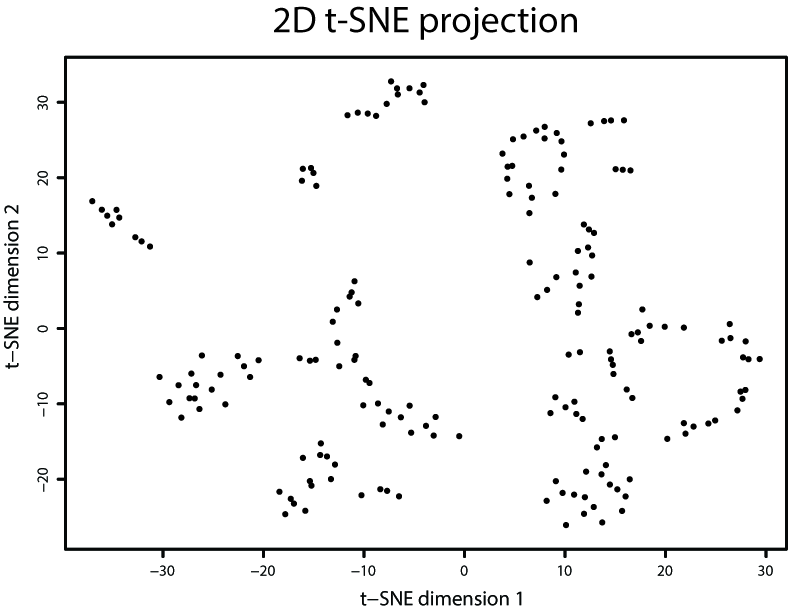

Supplement: Supplementary Figure 2 — Two-dimensional projection of the t-SNE analysis of the cells isolated from the systemic sclerosis (SSc) and healthy control (HC) skin. Cells were pooled together to facilitate cell subset identification. [file Image_2.TIF]

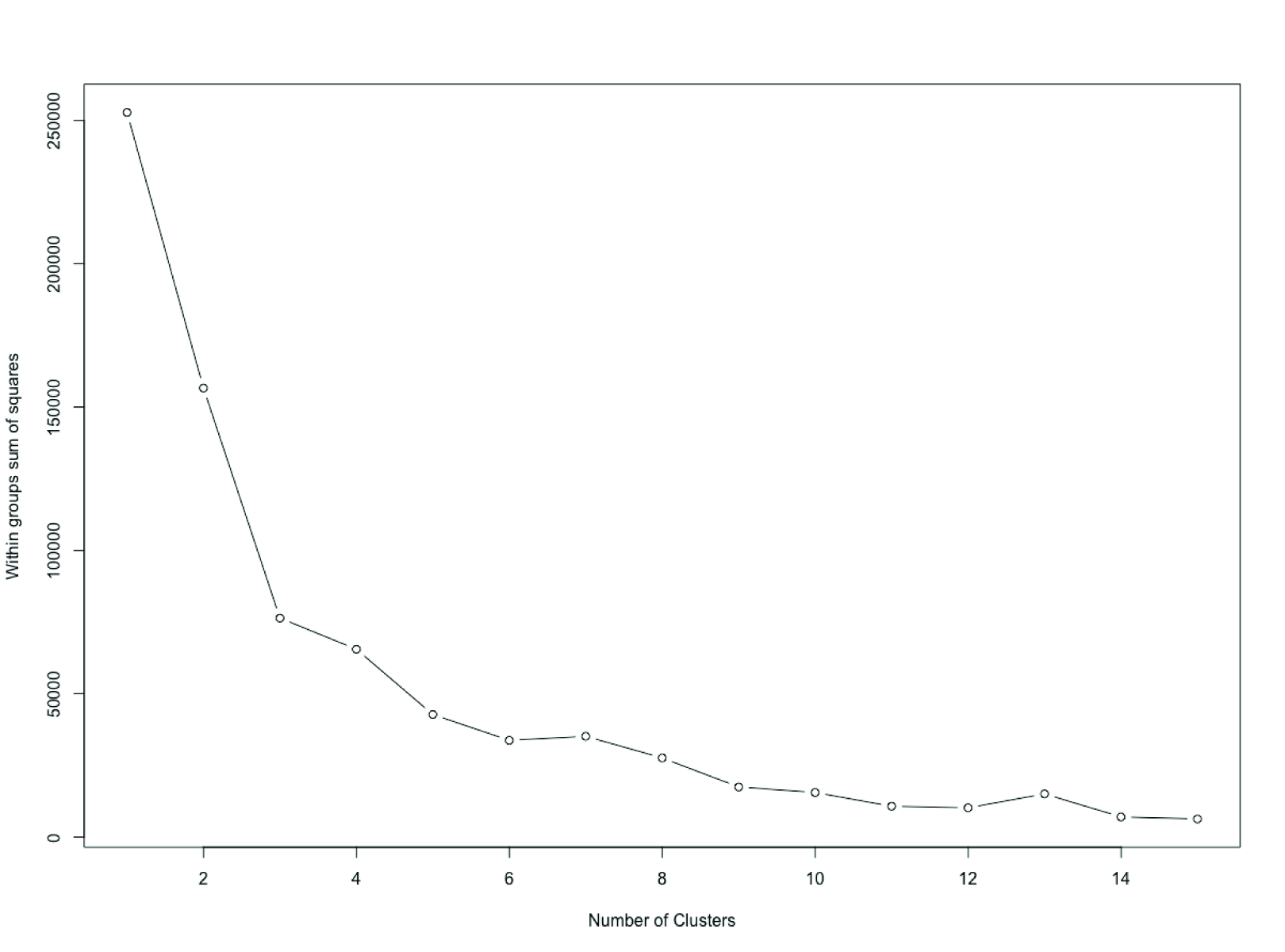

Supplement: Supplementary Figure 3 — The “elbow” criterion used to determine the optimal number of clusters to be used for the k-means clustering used in conjunction with the t-SNE analysis. [file Image_3.TIF]

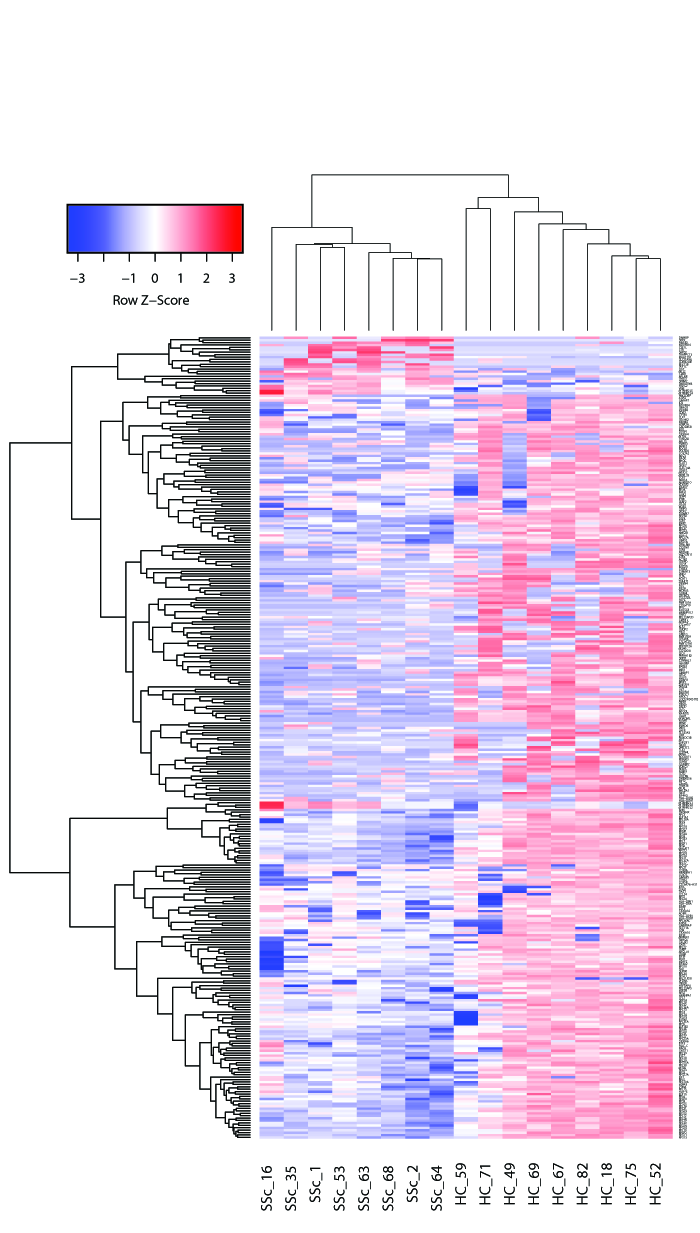

Supplement: Supplementary Figure 4 — Heatmap with hierarchical clustering of genes that were at least two-fold up-regulated or down-regulated in SSc endothelial cells compared to endothelial cells isolated from healthy skin. [file Image_4.TIF]
